# The impacts of faecal subsampling on microbial compositional profiling

**DOI:** 10.1186/s13104-022-05923-6

**Published:** 2022-02-14

**Authors:** Amanda J. Cox, Lily Hughes, Tiffanie M. Nelson, Kyle M. Hatton-Jones, Rebecca Ramsey, Allan W. Cripps, Nicholas P. West

**Affiliations:** 1 School of Medical Science, Griffith Health Centre (G40), Griffith University, Parklands Drive, Southport, QLD 4215 Australia; 2 Menzies Health Institute Queensland, Griffith University, Southport, QLD Australia; 3 Queensland Cyber Infrastructure Foundation Ltd Bioinformatics, St Lucia, QLD Australia; 4 School of Medicine, Griffith University, Southport, QLD Australia

**Keywords:** Gut microbiota, Faecal samples, Sub-sampling, 16s rRNA

## Abstract

**Objective:**

Despite the move to at-home, small-volume collection kits to facilitate large population-based studies of faecal microbial compositional profiling, there remains limited reporting on potential impacts of faecal subsampling approaches on compositional profiles. This study aimed to compare the microbial composition from faecal subsamples (< 5 g) collected from the beginning and end of a single bowel movement in ten otherwise healthy adults (6 female, 4 male; age: 24–55 years). Microbial composition was determined by V3–V4 16s rRNA sequencing and compared between subsamples.

**Results:**

There were no significant differences in OTU count (p = 0.32) or Shannon diversity index (p = 0.29) between the subsamples. Comparison of relative abundance for identified taxa revealed very few differences between subsamples. At the lower levels of taxonomic classification differences in abundance of the Bacillales (p = 0.02) and the Eubacteriaceae family (p = 0.03), and the Eubacterium genera (p = 0.03) were noted. The observation of consistent microbial compositional profiles between faecal subsamples from the beginning and end of a single bowel movement is an important outcome for study designs employing this approach to faecal sample collection. These findings provide assurance that use of a faecal subsample for microbial composition profiling is generally representative of the gut luminal contents more broadly.

**Supplementary Information:**

The online version contains supplementary material available at 10.1186/s13104-022-05923-6.

## Introduction

With exponential growth in the numbers of studies exploring the contribution of the gut microbiota to health and disease over the past decade, aligning sample collection approaches and analytical methods is crucial if the growing collection of outcomes are to be compared. Indeed, large numbers of studies have now assessed the impacts that differences in sample collection techniques (e.g. sample cups, swab-based collection kits), storage conditions (e.g. room temperature, immediate freezing, with/without stabilizing buffer), processing methods (e.g. with/without chemical and mechanical lysis), selection of 16s rRNA hypervariable regions for library preparation and sequencing, and data analysis pipelines (e.g. use of different reference databases) can have on microbial composition (see reviews, [1, 2]). The impact of these methodological factors on diversity metrics [3], detection of gram-positive bacteria [4], detection of anaerobic bacteria [5], and estimation of low-abundance/rare taxa [6] have all been acknowledged.

To support the recruitment of large, often geographically distant cohorts, earlier sample collection methods suggesting homogenization of the entire bowel movement and subsampling of the homogenate for subsequent compositional profiling [7] have fallen out of favor in preference for in-home collection kits. These methods require volunteers to sample a small amount of faecal material, but standardization of subsampling in the context of the complete bowel movement is rarely stipulated in available protocols. Given consideration of the implications of other aspects of sample collection/storage/handling on overall microbial composition, it is surprising that the potential impacts of faecal subsampling have been less readily documented. Of the few studies we are aware of, findings are inconsistent; Gorzelak et al. [8] have previously reported differences in the abundance of a number of key taxa in subsamples taken from outer and inner stool microenvironments, whereas Santiago et al. [9] reported largely similar composition from outer and inner stool microenvironments. Conscious of the apparent lack of empirical data supporting consistent microbial composition, or otherwise, from faecal subsamples and the considerable practical implications of such observations, the aim of this study was to compare the microbial composition from faecal subsamples collected from the beginning and end of a single bowel movement.

## Main text

### Methods

This study involved the analysis of faecal material collected as part of the AussieGut™ program for the determination of faecal microbial composition. Individuals (n = 10; 6 female, 4 male; age: 24–55 years) were instructed to collect a small (< 5 g) faecal subsample from the beginning and end of a single bowel movement using provided faecal collection kits. Collection kits included a 70 mL faecal collection cup with scooped lid (Sarstedt, SA, Australia) and flushable collection paper (Eiken Chemical, Tokyo, Japan). Samples were to be free of water and urine. Faecal samples were returned to the laboratory within 24 h of collection and stored at − 80 °C until analysis. Individuals provided written informed consent prior to participation and ethical approval was provided by the Griffith University Human Research Ethics Committee (ref#: MED/19/15/HREC).

Thawed faecal samples were initially homogenized in phosphate buffered saline, followed by repeated cycles of chemical and mechanical (bead beating) lysis and subsequent DNA extraction using a commercially available kit (Qiagen, Hilden, Germany) as has been previously reported [10]. The V3-V4 region of the microbial 16s rRNA marker gene was amplified using universal primers (F: 5′-CCTACGGGNGGCWGCAG-3′; R: 5′-GACTACHVGGGTATCTAATCC-3′) [11], and polymerase chain reaction products sequenced on an Illumina MiSeq system (Illumina, California, USA) by a commercial provider (Macrogen, Seoul, South Korea). Clustering of sequence reads into operational taxonomic units (OTU) at 97% identity level was achieved using the Quantitative Insights in Microbial Ecology (QIIME) Suite [12]. The ChimeraSlayer program was used to remove chimaeras from aligned OTU and the FastTreeMP tool generated a phylogenetic tree. Taxonomic identity assignment was performed using a reference-based approach with the NCBI database of 16S rRNA gene sequences.

Identified microbial taxa were considered as prevalent (present in greater than 75% of the samples) or not-prevalent (present in less than 25% of the samples). A principle components analysis (PCoA) was used to explore the difference in the global microbial composition between the subsamples. The relative abundance of prevalent taxa were compared between subsamples using a paired-sample t-test or a Wilcoxon matched-pair signed rank test. Statistical significance was accepted at p < 0.05.

## Results

An average of 27,976 ± 4955 reads per sample were used for taxa assignment. The total OTU count between subsamples did not differ (p = 0.32), with approximately 240 OTU identified (Table 1). Similarly, the Shannon diversity index was consistent between subsamples (p = 0.29). At the phyla level, five phyla were prevalent; the relative abundance of these key phyla were consistent between the subsamples (Table 1). The Cyanobacteria (detected in a single sample only) and Tenericutes phyla (detected in both samples from two individuals) were considered not prevalent in the sample set and persisted at low relative abundance (< 0.05%).

**Table 1:** Diversity metrics and relative abundance data for prevalent bacterial phyla between faecal subsamples. Data are presented as mean ± SD (median; range)

	Sub-sample 1	Sub-sample 2	p-value
Diversity metrics			
OTU count	243 ± 75 (243; 140–362)	237 ± 71 (235; 135–360)	0.32
Shannon Index	4.69 ± 0.69 (4.83; 3.71–5.50)	4.77 ± 0.69 (4.88; 3.70–5.75)	0.29
Phyla (relative abundance)			
Actinobacteria	0.41 ± 0.25 (0.41; 0.02–0.81)	0.42 ± 0.32 (0.29; 0.01–0.98)	0.80
Bacteroidetes	30.9 ± 12.1 (29.8; 15.4–49.0)	29.7 ± 11.2 (28.3; 16.8–47.1)	0.51
Firmicutes	63.2 ± 11.3 (58.8; 50.0–81.3)	65.7 ± 10.5 (65.7; 52.0–81.2)	0.35
Proteobacteria	1.78 ± 1.85 (1.03; 0.19–6.20)	1.22 ± 0.80 (0.99; 0.24–2.62)	0.92^a^
Verrucomicrobia	3.57 ± 5.83 (1.37; 0–18.0)	2.78 ± 4.14 (1.06; 0.0–12.6)	0.59^a^

^a^p value from Wilcoxon sign-ranked test

**Fig. 1:**
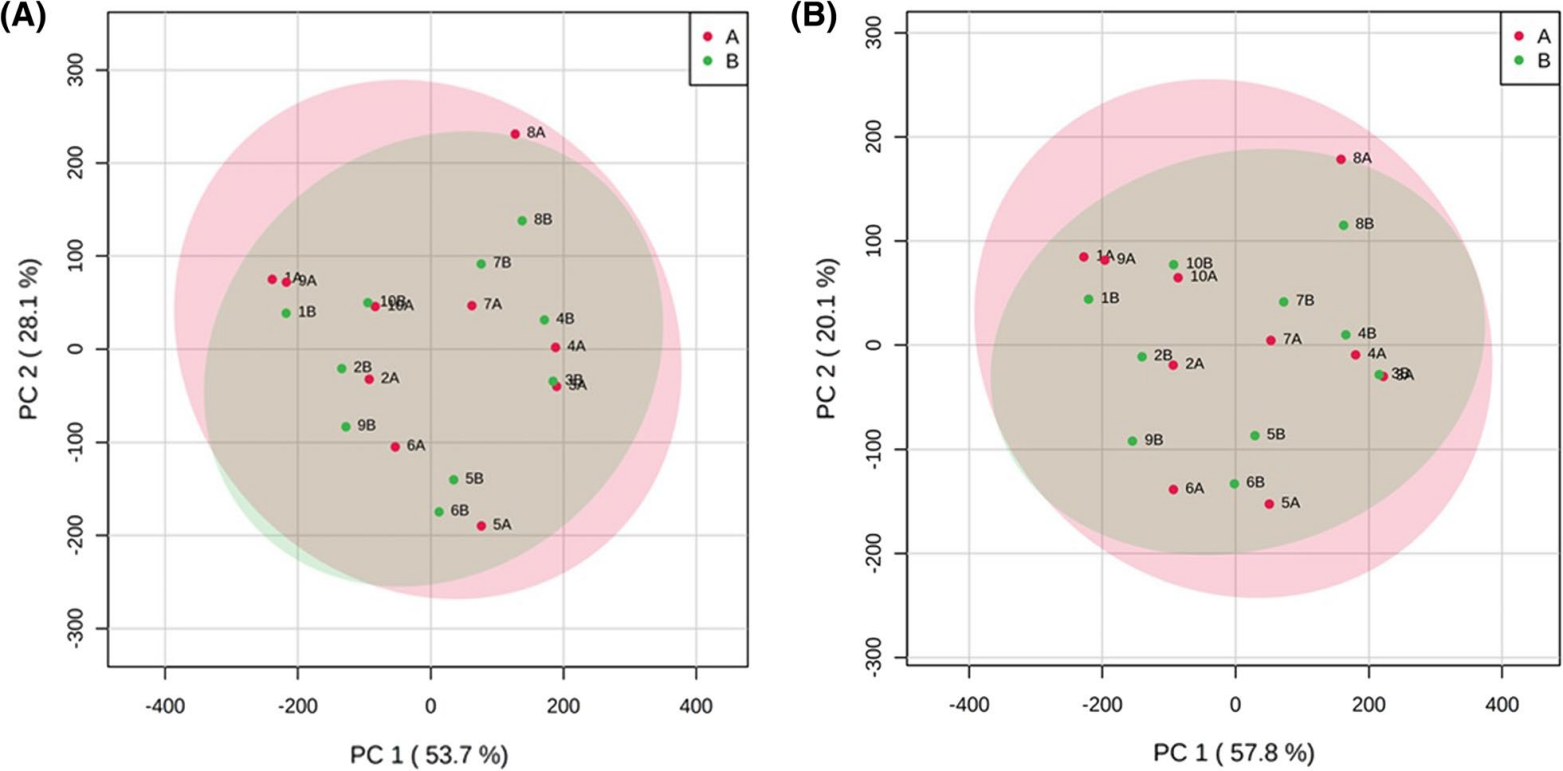
Principle component analysis for (**A**) prevalent families (**B**) prevalent genera

**Fig. 2:**
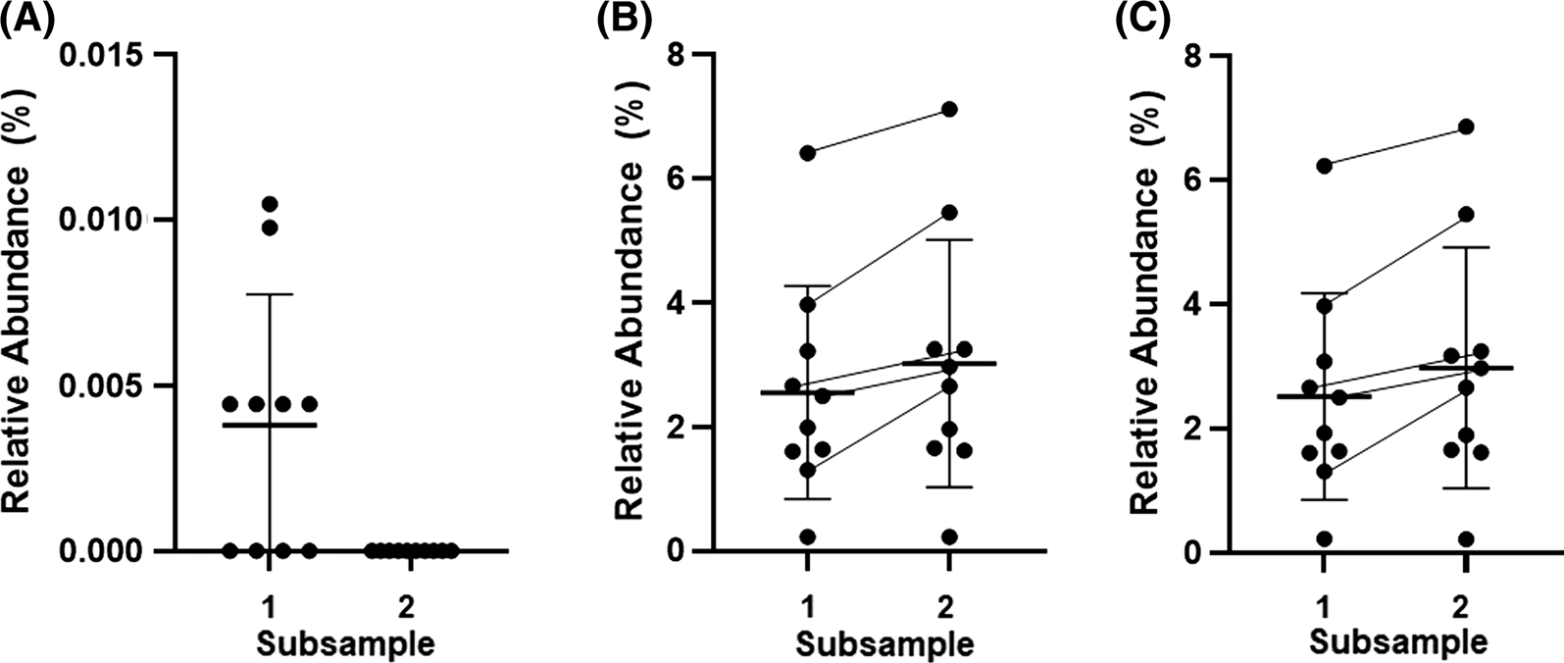
Relative abundance of (**A**) unclassified Bacillales (**B**) Eubacteriaceae family (**C**) Eubacterium genera between subsample 1 and subsample 2

At the Family level, 44 unique bacterial families were identified; seven of these families were not prevalent within the sample set (Additional file 1: Table S1). A PCoA using the prevalent families indicated that the microbial composition was generally similar between the subsamples (Fig. 1A). Further consideration of the prevalent families indicated that 35 of the 37 families had a consistent relative abundance between the subsamples; the relative abundance of the unclassified Bacillales (p = 0.02) and the Eubacteriaceae family (p = 0.03) differed between the subsamples. For the unclassified Bacillales, this was accounted for by the detection of the taxa in five of the participants’ subsample 1, but in none of the participants’ subsample 2 (Fig. 2A). For the Eubacteriaceae family, the difference between the two subsamples was driven by a greater (10%—twofold) relative abundance in subsample 2 for five participants (Fig. 2B).

At the Genus level, 109 unique bacterial genera were identified; 31 of these genera were not prevalent within the sample set (Additional file 2: Table S2). A PCoA using the prevalent genera indicated that the microbial composition was generally similar between the subsamples (Fig. 1B). Further consideration of the prevalent genera indicated that 77 of the 78 prevalent genera had a consistent relative abundance between the subsamples; the relative abundance of the Eubacterium genera (p = 0.03) differed between the subsamples. This difference was driven by a greater (10%—twofold) relative abundance in subsample 2 for five participants (Fig. 2C).

## Discussion

Efforts to better understand the implications of various methodological choices on the study of microbial composition are well documented [1, 2]. However, the potential for variability in faecal subsamples from a single bowel movement appears less well characterized. With the move to greater use of at-home, low-volume faecal collection kits to facilitate large population-based studies, empirical data to support consistent microbial composition between faecal subsamples from a single bowel movement is crucial. This study was able to demonstrate that global diversity metrics and composition to the genus level were broadly consistent between faecal subsamples collected from the beginning and end of a single bowel movement in otherwise healthy adults.

Quantification of the inherent variability in microbial composition, both within a single bowel movement and within consecutive bowel movements (in the absence of other intervention or dietary modification), appears relatively limited. In contrast, attempts to document spatial heterogeneity in microbial composition along the gastrointestinal tract are more common [13] and suggest differences in composition may persist even between relatively close anatomical sampling sites such as the sigmoid colon and rectum [14]. Our study has been able to help clarify if such differences carry over to luminal contents and create distinct niches within a single bowel movement. Our findings of broadly consistent microbial diversity and composition between faecal subsamples are also in agreement with the prior report from Santiago et al. [9] who found microbial composition was largely similar between outer and inner stool microenvironments. We do make note of the tendency for increased relative abundance of the anaerobic Eubacterium genera, in the subsample collected from the end of the bowel movement among half of the participants, however it is unclear why this trend was evident for this one genera only and other members of the Clostridia class were not similarly impacted.

## Limitations

We acknowledge that the study is not without its limitations, employing a modest sample size and collection of subsamples from the beginning and end of the bowel movement only. Aware of the limitation of 16s methodologies for species level identification [15], we chose to report to the genus level only and so it is unclear if the largely consistent compositional profiles between subsamples extend beyond the genus level and include rare taxa. In addition, our study recruited otherwise healthy adults only and it is not clear if findings of consistent compositional features between subsamples would also be observed in particular disease states where skewed microbial composition may be anticipated. However, the outcomes do provide assurance that use of methodologies which employ collection of a faecal subsample can provide representation of the microbial composition from the gut luminal contents more broadly.

## Supplementary Information

**Additional file 1: Table S1**. Prevalence and relative abundance of bacterial families from five key phyla for paired faecal subsamples.

**Additional file 2: Table S2**. Prevalence and relative abundance of bacterial genera from five key phyla for paired fecal subsamples.

## Data Availability

The datasets used and/or analysed during the current study are available from the corresponding author on reasonable request.

## Abbreviations

OTU: Operational taxonomic units
QIIME: Quantitative Insights in Microbial Ecology
PCoA: Principle components analysis
