# Supplementary material for: The impacts of faecal subsampling on microbial compositional profiling
Source: BMC Res Notes. 2022 Feb 14;15:49. doi: 10.1186/s13104-022-05923-6 (PMC8842933; doi:10.1186/s13104-022-05923-6)
Supplement: Supplementary file 1 — Additional file 1: Table S1. Prevalence and relative abundance of bacterial families from five key phyla for paired faecal subsamples. [file 13104_2022_5923_MOESM1_ESM.pdf]

Supplementary Table 1

Prevalence and relative abundance of bacterial families from five key phyla for paired faecal subsamples.

	Taxa Detected (# participants)		Mean ± SD (median; range)		
	Sub-sample 1 (/10)	Sub-sample 2 (/10)	Sub-sample 1	Sub-sample 2	p-value*
**Actinobacteria**					
unclassified	10	9	0.074 ± 0.079 (0.057; 0.009-0.269)	0.089 ± 0.111 (0.045; 0.0-0.351)	0.72
Micrococcaceae	4	4	0.002 ± 0.003 (0.0; 0.0-0.010)	0004 ± 0.008 (0.0; 0.0-0.024)	0.35
Actinomycetaceae	4	1	0.002 ± 0.002 (0.0; 0.0-0.007)	0.0004 ± 0.001 (0.0; 0.0-0.004)	0.23
Bifidobacteriaceae	9	9	0.247 ± 0.238 (0.132; 0.0-0.685)	0.241 ± 0.264 (0.160; 0.0-0.824)	0.86
†Porphyromonadaceae	1	1	-	-	-
Coriobacteriales, unclassified	4	3	0.005 ± 0.008 (0.0; 0.0-0.024)	0.002 ± 0.005 (0.0; 0.0-0.016)	0.07
Coriobacteriaceae	8	8	0.082 ± 0.091 (0.050; 0.0-0.270)	0.081 ± 0.095 (0.0; 0.0-0.258)	1.00
**Bacteroidetes**					
Flavobacteriaceae	2	3	0.009 ± 0.025 (0.0; 0.0-0.080)	0.012 ± 0.033 (0.0; 0.0-0.107)	0.11
Bacteroidales, unclassified	10	10	4.26 ± 3.83 (2.38; 0.290-10.97)	4.06 ± 3.82 (2.310; 0.367-11.13)	0.88
Bacteroidaceae	10	10	22.71 ± 13.41 (20.70; 3.41-42.66)	21.61 ± 12.01 (17.75; 3.72-40.57)	0.50^{t}
Prevotellaceae	7	6	0.501 ± 0.776 (0.048; 0.0-1.83)	0.660 ± 1.15 (0.072; 0.0-3.34)	0.40
Rikenellaceae	10	10	3.45 ± 2.66 (3.75; 0.058-8.98)	3.36 ± 2.74 (3.27; 0.028-8.55)	0.75^{t}
**Firmicutes**					
unclassified	3	4	0.006 ±0.016 (0.0; 0.0-0.052)	0.006 ± 0.011 (0.0; 0.0-0.037)	0.89
Erysipelotrichaceae	10	10	0.557 ± 0.603 (0.418; 0.133-2.21)	0.673 ± 0.754 (0.368; 0.168-2.60)	0.20
Bacillales, unclassified	5	0	0.004 ± 0.004 (0.004; 0.0-0.010)	0.0 ± 0.0 (0; 0.0-0.0)	0.02

Carnobacteriaceae	3	2	0.002 ± 0.003 (0.0; 0.0-0.010)	0.001 ± 0.002 (0.0; 0.0-0.006)	0.72
†Enterococcaceae	1	1	-	-	-
Lactobacillaceae	6	5	0.101 ± 0.175 (0.007; 0.0-0.450)	0.124 ± 0.228 (0.002; 0.0-0.655)	0.92
Streptococcaceae	10	10	0.200 ± 0.362 (0.052; 0.003-1.20)	0.219 ± 0.401 (0.058; 0.019-1.33)	0.20
Clostridiales, unclassified	10	10	6.64 ± 6.06 (5.77; 0.39-16.99)	6.76 ± 6.02 (5.41; 0.23-16.17)	0.82^{t}
Catabacteriaceae	4	4	0.003 ± 0.004 (0.0; 0.0-0.013)	0.002 ± 0.002 (0.0; 0.0-0.006)	0.75
Christensenellaceae	9	8	0.888 ± 1.95 (0.127; 0.0-6.33)	0.970 ± 2.30 (0.129; 0.0-7.46)	0.44
Clostridiaceae	10	9	0.466 ± 0.383 (0.309; 0.015-1.13)	0.550 ± 0.404 (0.454; 0.0-1.17)	0.20^{t}
Clostridiales Family XIII. Incertae Sedis	9	9	0.171 ± 0.190 (0.125; 0.0-0.629)	0.175 ± 0.211 (0.114; 0.0-0.719)	0.86
Eubacteriaceae	10	10	2.56 ± 1.71 (2.25; 0.23-6.42)	3.02 ± 1.99 (2.82; 0.23-7.12)	0.03^{t}
Lachnospiraceae	10	10	15.45 ± 5.63 (15.19; 5.96-23.89)	15.64 ± 6.76 (15.01; 7.12-27.56)	0.86^{t}
Oscillospiraceae	10	10	2.62 ± 2.44 (1.63; 0.153-6.99)	2.71 ± 2.60 (1.40; 0.204-7.49)	0.60^{t}
Peptococcaceae	6	7	0.027 ± 0.035 (0.016; 0.0-0.112)	0.028 ± 0.041 (0.015; 0.0-0.133)	1.00
Peptostreptococcaceae	10	10	0.173 ± 0.194 (0.110; 0.004-0.664)	0.214 ± 0.200 (0.137; 0.012-0.556)	0.45
Ruminococcaceae	10	10	29.01 ± 10.15 (28.22; 11.84-48.92)	30.95 ± 8.01 (29.87; 19.37-44.27)	0.36^{t}
Acidaminococcaceae	9	8	1.38 ± 2.20 0.361; 0.0-1.04)	1.13 ± 1.81 (0.337; 0.0-5.30)	0.37
Veillonellaceae	10	9	2.12 ± 2.53 (1.33; 0.003-6.90)	1.72 ± 2.13 (1.18; 0.0-6.27)	0.58
†Selenomonadaceae	1	2	-	-	-
**Proteobacteria**					
†Kiloniellaceae	1	1	-	-	-
Rhodospirillaceae	3	6	0.126 ± 0.325 (0.0; 0.0-1.04)	0.157 ± 0.441 (0.007; 0.0-1.41)	0.30
Oxalobacteraceae	6	6	0.015 ± 0.017 (0.010; 0.0-0.045)	0.012 ± 0.012 (0.009; 0.0-2.18)	0.33^{t}
Sutterellaceae	9	9	1.24 ± 1.82 (0.479; 0.0-5.73)	0.652 ± 0.642 (0.469; 0.0-2.18)	0.77

Desulfovibrionaceae	10	10	0.183 ± 0.162 (0.140; 0.003-0.431)	0.165 ± 0.167 (0.080; 0.016-0.422)	0.45
Enterobacteriaceae	8	9	0.143 ± 0.294 (0.049; 0.0-0.970)	0.100 ± 0.104 (0.083; 0.0-0.292)	0.44
Pasteurellaceae	8	8	0.061 ± 0.058 (0.051; 0.0-0.151)	0.122 ± 0.233 (0.049; 0.0-0.770)	0.89
†Acholeplasmataceae	1	2	-	-	-
†Spiroplasmataceae	2	2	-	-	-
**Verrucomicrobia**					
unclassified	8	9	3.57 ± 5.83 (1.346; 0.0-18.00)	2.77 ± 4.14(1.061; 0.0-12.63)	0.59
†Verrucomicrobiaceae	1	1	-	-	-

† taxa considered as not prevalent (i.e. present in less than 25% of samples), summary statistics and between group comparisons not performed; *p-value from Wilcoxon signed rank test unless specified; ^{t}p-value from paired-sample t-test
