# Supplementary material for: The impacts of faecal subsampling on microbial compositional profiling
Source: BMC Res Notes. 2022 Feb 14;15:49. doi: 10.1186/s13104-022-05923-6 (PMC8842933; doi:10.1186/s13104-022-05923-6)
Supplement: Supplementary file 2 — Additional file 2: Table S2. Prevalence and relative abundance of bacterial genera from five key phyla for paired fecal subsamples. [file 13104_2022_5923_MOESM2_ESM.pdf]

Supplementary Table 2

Prevalence and relative abundance of bacterial genera from five key phyla for paired fecal subsamples.

	Taxa Detected (# participants)		Mean SD (median; range)		
	Sample 1 (/10)	Sample 2 (/10)	Sample 1	Sample 2	p-value*
**Actinobacteria**					
unclassified	6	4	0.033 ± 0.076 (0.004; 0.0-0.245)	0.038 ± 0.093 (0.0; 0.0-0.298)	0.75
†Adlercreutzia	2	1	-	-	-
Asaccharobacter	6	5	0.007 ± 0.010 (0.005; 0.0-0.033)	0.007 ± 0.012 (0.002; 0.0-0.038)	1.00
Eggerthella	5	6	0.027 ± 0.039 (0.004; 0.0-0.098)	0.036 ± 0.061 (0.004; 0.0-0.171)	0.60
Slackia	4	2	0.006 ± 0.011 (0.0; 0.0-0.035)	0.007 ± 0.016 (0.0; 0.0-0.049)	0.72
Rothia	3	4	0.002 ± 0.003 (0.0; 0.0-0.010)	0.004 ± 0.008 (0.0; 0.0-0.024)	0.14
†Actinomyces	3	1	-	-	-
Bifidobacterium	9	9	0.247 ± 0.238 (0.132; 0.0-0.685)	0.241 ± 0.264 (0.160; 0.0-0.824)	0.86
Atopobium	4	3	0.005 ± 0.008 (0.0; 0.0-0.024)	0.002 ± 0.005 (0.0-; 0.0-0.016)	0.07
Coriobacteriaceae, unclassified	5	4	0.013 ± 0.031 (0.002; 0.0-0.101)	0.013 ± 0.029 (0.0; 0.0-0.090)	1.00
Collinsella	8	7	0.068 ± 0.084 (0.045; 0.0-0.270)	0.068 ± 0.023 (0.029; 0.0-0.258)	0.89
**Bacteroidetes**					
†Coenonia	2	2	-	-	-
Bacteroidales, unclassified	7	8	2.43 ± 3.17 (0.760; 0.0-8.77)	2.38 ± 3.20 (0.584; 0.0-8.66)	0.48
Barnesiella	10	9	1.08 ± 1.41 (0.295; 0.003-3.72)	1.00 ± 1.25 (0.439; 0.0-3.74)	0.76
Butyricimonas	6	6	0.054 ± 0.074 (0.018; 0.0-0.189)	0.043 ± 0.066 (0.008; 0.0-0.183)	0.46
Odoribacter	7	7	0.126 ± 0.147 (0.064; 0.0-0.371)	0.102 ± 0.118 (0.068; 0.0-0.368)	0.74

Parabacteroides	8	7	0.579 ± 1.20 (0.216; 0.0-0.581)	0.547 ± 1.24 (0.185; 0.0-4.04)	0.40
Bacteroides	10	10	22.71 ± 13.41 (20.70; 3.41-42.66)	21.61 ± 12.01 (17.75; 3.72-40.57)	0.50[t]
†Porphyromonadaceae, unclassified	1	1	-	-	-
†Paraprevotella	2	2	-	-	-
Prevotella	6	5	0.386 ± 0.732 (0.013; 0.0-1.83)	0.489 ± 1.05 (0.032; 0.0-3.34)	0.60
Rikenellaceae, unclassified	3	3	0.235 ± 0.494 (0.0; 0.0-1.26)	0.100 ± 0.260 (0.0; 0.0-0.824)	0.11
Alistipes	10	10	3.22 ± 2.50 (3.75; 0.058-7.72)	3.26 ± 2.60 (3.27; 0.028-7.73)	0.72
**Firmicutes**					
Sporanaerobacter	2	3	0.006 ± 0.16 (0.0; 0.0-0.052)	0.005 ± 0.012 (0.0; 0.0-0.037)	1.00
Erysipelotrichaceae, unclassified	10	10	0.478 ± 0.614 (0.318; 0.078-2.17)	0.588 ± 0.752 (0.261; 0.128-2.52)	0.20
†Catenibacterium	1	1	-	-	-
Holdemania	8	8	0.019 ± 0.023 (0.012; 0.0-0.065)	0.019 ± 0.015 (0.020; 0.0-0.040)	0.76
†Solobacterium	0	1	-	-	-
Turicibacter	5	8	0.039 ± 0.090 (0.007; 0.0-0.293)	0.039 ± 0.079 (0.009; 0.0-0.261)	0.78
†Bacillales, unclassified	1	0	-	-	-
†Gemella	2	0	-	-	-
†Granulicatella	2	2	-	-	-
†Enterococcus	0	1	-	-	-
Lactobacillus	6	5	0.101 ± 0.174 (0.074; 0.0-0.450)	0.124 ± 0.228 (0.002; 0.0-0.655)	0.92
Streptococcus	10	10	0.200 ± 0.362 (0.052; 0.003-1.20)	0.219 ± 0.399 (0.058; 0.019-1.33)	0.20
Clostridiales, unclassified	10	10	5.31 ± 5.89 (1.73; 0.389-16.24)	5.47 ± 5.83 (1.80; 0.23-15.45)	0.89
Gracilibacter	8	8	1.31 ± 1.64 (0.611; 0.0-4.45)	1.28 ± 1.45 (0.674; 0.0-3.96)	0.89
Howardella	3	3	0.020 ± 0.037 (0.0; 0.0-0.112)	0.015 ± 0.036 (0.0; 0.0-0.113)	0.29
Catabacter	3	4	0.002 ± 0.004 (0.0; 0.0-0.013)	0.002 ± 0.002 (0.0; 0.0-0.006)	1.00

Christensenella	9	8	0.888 ± 1.95 (0.127; 0.0-6.33)	0.970 ± 2.30 (0.129; 0.0-7.46)	0.44
Clostridiaceae, unclassified	7	7	0.081 ± 0.143 (0.014; 0.0-0.429)	0.053 ± 0.104 (0.008; 0.008-0.329)	0.09
Butyricicoccus	2	4	0.013 ± 0.031 (0.0; 0.0-0.093)	0.011 ± 0.022 (0.0; 0.0-0.070)	0.47
Caloramator	8	8	0.169 ± 0.184 (0.150; 0.0-0.654)	0.184 ± 0.188 (0.133; 0.0-0.635)	1.00
Clostridium	10	9	0.195 ± 0.268 (0.119; 0.003-0.859)	0.279 ± 0.372 (0.135; 0.0-0.966)	0.46
Lutispora	6	8	0.008 ± 0.011 (0.005; 0.0-0.029)	0.018 ± 0.024 (0.010; 0.0-0.071)	0.09
†Thermotalea	0	1	-	-	-
†Tindallia	1	1	-	-	-
Clostridiales Family XIII. Incertae Sedis	9	8	0.095 ± 0.096 (0.068; 0.0-0.241)	0.081 ± 0.093 (0.046; 0.0-0.284)	0.21
Anaerovorax	8	8	0.076 ± 0.130 (0.034; 0.0-0.430)	0.094 ± 0.164 (0.056; 0.0-0.551)	0.44
Eubacteriaceae, unclassified	6	7	0.025 ± 0.059 (0.003; 0.0-0.191)	0.032 ± 0.084 (0.006; 0.0-0.263)	0.34
†Alkalibaculum	1	0	-	-	-
Eubacterium	10	10	2.51 ± 1.66 (2.22; 0.224-6.23)	2.98 ± 1.94 (2.82; 0.219-6.86)	0.03[t]
†Garciella	2	2	-	-	-
Lachnospiraceae, unclassified	10	10	7.73 ± 3.80 (7.18; 2.86-17.12)	7.41 ± 3.92 (6.35; 3.21-16.63)	0.24
†Anaerosporobacter	2	1	-	-	-
Anaerostipes	10	10	0.158 ± 0.204 (0.071; 0.027-0.681)	0.190 ± 0.245 (0.062; 0.007-0.682)	0.58
Blautia	10	10	2.19 ± 1.40 (1.89; 0.563-4.60)	2.52 ± 2.01 (1.92; 0.527-6.81)	0.34[t]
†Butyrivibrio	2	2	-	-	-
Coprococcus	9	9	2.53 ± 3.51 (0.758; 0.0-9.39)	2.50 ± 3.33 (0.773; 0.0-8.36)	0.68
Dorea	8	8	0.279 ± 0.276 (0.219; 0.0-0.843)	0.264 ± 0.304 (0.172; 0.0-0.991)	0.78
Hespellia	7	7	0.068 ± 0.080 (0.036; 0.0-0.203)	0.056 ± 0.066 (0.036; 0.0-0.177)	0.09
Lachnobacterium	9	9	0.226 ± 0.305 (0.117; 0.0-0.912)	0.280 ± 0.419 (0.074; 0.0-1.099)	0.31
Lachnospira	9	10	0.907 ± 1.43 (0.162; 0.0-4.43)	0.723 ± 0.980 (0.117; 0.013-2.52)	0.86

Parasporobacterium	6	6	0.024 ± 0.034 (0.008; 0.0-0.084)	0.011 ± 0.014 (0.005; 0.0-0.043)	0.17
Pseudobutyrivibrio	7	7	0.050 ± 0.056 (0.033; 0.0-0.156)	0.029 ± 0.026 (0.030; 0.0-0.077)	0.14^{t}
Robinsoniella	3	3	0.007 ± 0.013 (0.0; 0.0-0.039)	0.006 ± 0.014 (0.0; 0.0-0.045)	0.47
Roseburia	10	10	1.19 ± 1.48 (0.307; 0.008-4.64)	1.49 ± 1.83 (0.453; 0.003-5.210)	0.17
Oscillibacter	10	10	2.62 ± 2.44 (1.63; 0.153-6.99)	2.71 ± 2.60 (1.40; 0.20-7.49)	0.60^{t}
†Peptococcaceae, unclassified	2	1	-	-	-
†Desulfitobacterium	0	1	-	-	-
Desulfonispora	3	3	0.002 ± 0.004 (0.0; 0.0-0.013)	0.003 ± 0.007 (0.0; 0.0-0.021)	0.59
†Desulfosporosinus	2	2	-	-	-
Desulfotomaculum	3	3	0.006 ± 0.013 (0.0; 0.0-0.042)	0.004 ± 0.009 (0.0; 0.0-0.027)	0.18
Peptococcus	5	6	0.016 ± 0.031 (0.0; 0.0-0.099)	0.018 ± 0.037 (0.004; 0.0-0.120)	0.40
Peptostreptococcaceae, unclassified	10	10	0.173 ± 0.195 (0.110; 0.004-0.664)	0.214 ± 0.200 (0.137; 0.012-0.556)	0.45
†Peptostreptococcus	2	1	-	-	-
Ruminococcaceae, unclassified	9	9	3.89 ± 3.50 (3.14; 0.0-9.55)	3.91 ± 3.50 (2.73; 0.0-10.12)	0.97^{t}
Acetanaerobacterium	9	6	0.149 ± 0.225 (0.018; 0.0-0.546)	0.160 ± 0.245 (0.008; 0.0-0.597)	0.64
Acetivibrio	9	7	0.204 ± 0.222 (0.101; 0.0-0.554)	0.206 ± 0.274 (0.056; 0.0-0.809)	0.44
Anaerofilum	4	3	0.002 ± 0.003 (0.0; 0.0-0.009)	0.003 ± 0.005 (0.0; 0.0-0.014)	0.92
Anaerotruncus	7	9	0.021 ± 0.020 (0.022; 0.0-0.051)	0.030 ± 0.025 (0.022; 0.0-0.074)	0.08
Ethanoligenens	7	6	0.055 ± 0.108 (0.013; 0.0-0.351)	0.071 ± 0.142 (0.007; 0.0-0.458)	0.14
Faecalibacterium	10	10	20.75 ± 8.65 (19.98; 5.21-35.21)	21.58 ± 7.87 (22.04; 9.01-35.08)	0.72^{t}
Gemmiger	9	10	1.81 ± 1.46 (1.58; 0.0-4.44)	1.94 ± 1.52 (1.86; 0.006-5.10)	0.43^{t}
Hydrogenoanaerobacterium	2	3	0.003 ± 0.006 (0.0; 0.0-0.016)	0.003 ± 0.008 (0.0; 0.0-0.026)	0.72
†Papillibacter	2	1	-	-	-
Ruminococcus	10	10	1.05 ± 0.636 (0.812; 0.239-2.21)	1.91 ± 2.43 (1.24; 0.217-8.58)	0.39

Sporobacter	9	9	0.442 ± 0.745 (0.076; 0.0-2.11)	0.492 ± 0.763 (0.066; 0.0-1.88)	0.67
†Subdoligranulum	0	1	-	-	-
†Acidaminococcus	2	2	-	-	-
Phascolarctobacterium	8	8	1.10 ± 1.79 (0.361; 0.0-5.63)	0.853 ± 1.23 (0.337; 0.0-3.54)	0.31
Dialister	6	6	2.07 ± 2.54 (1.30; 0.0-6.87)	1.68 ± 2.14 (1.16; 0.0-6.23)	0.75
Veillonella	9	8	0.044 ± 0.053 (0.028; 0.0-0.156)	0.037 ± 0.060 (0.013; 0.0-0.197)	0.84
†Megamonas	1	1	-	-	-
†Propionispira	0	1	-	-	-
**Proteobacteria**					
†Kiloniella	1	1	-	-	-
†Rhodospirillaceae, unclassified	1	1	-	-	-
Novispirillum	2	3	0.021 ± 0.055 (0.0; 0.0-0.173)	0.013 ± 0.027 (0.0; 0.0-0.082)	1.00
†Rhodospirillum	1	3	-	-	-
Oxalobacter	6	6	0.015 ± 0.017 (0.010; 0.0-0.045)	0.012 ± 0.012 (0.009; 0.0-0.033)	0.35[t]
Sutterellaceae, unclassified	9	9	1.16 ± 1.85 (0.377; 0.0-5.69)	0.574 ± 0.971 (0.380; 0.0-2.18)	0.95
Sutterella	4	4	0.074 ± 0.149 (0.0; 0.0-0.476)	0.078 ± 0.185 (0.0; 0.0-0.594)	1.00
†Desulfomicrobium	1	1	-	-	-
Desulfovibrio	9	9	0.180 ± 0.166 (0.140; 0.000-0.431)	0.161 ± 0.171 (0.080; 0.0-0.422)	0.34
Escherichia	7	8	0.130 ± 0.299 (0.026; 0.0-0.970)	0.084 ± 0.107 (0.038; 0.0-0.292)	0.29
Klebsiella	5	5	0.013 ± 0.024 (0.002; 0.0-0.065)	0.016 ± 0.028 (0.0; 0.003-0.083)	0.60
Haemophilus	8	8	0.061 ± 0.058 (0.051; 0.0-0.151)	0.122 ± 0.233 (0.049; 0.0-0.770)	0.89
**Verrucomicrobia**					
Akkermansia	8	9	3.57 ± 5.83 (1.346; 0.0-18.003)	2.77 ± 4.14 (1.061; 0.0-12.627)	0.59
†Fucophilus	1	1	-	-	-

† taxa not prevalent (i.e. present in less than 25% of samples), summary statistics and between group comparisons not performed; *p-value from Wilcoxon signed rank test unless specified; [t]p-value from paired-sample t-test
